# The use of telehealth to overcome barriers to mental health services faced by young people from Afro-Caribbean backgrounds in England during the COVID-19 pandemic

**DOI:** 10.7189/jogh.11.03040

**Published:** 2021-03-01

**Authors:** Aditi Rajgopal, C Raymond Li, Shahini Shah, Shyam Sundar Budhathoki

**Affiliations:** School of Public Health, Faculty of Medicine, Imperial College London, London, UK

Mental health in young people is a pertinent issue. In England, one in eight 5 to 19 year-olds have a diagnosable mental health condition, but only a third are accessing treatment [1]. Anxiety disorders are the most common disorders amongst 11 to 19 year-olds [2]. Research investigating associations between mental health in children and ethnicity is lacking, with a thirteen-year gap between the last two national Mental Health of Children and Young People Surveys, published in 2005 and 2018. However, there is evidence suggesting lower utilisation of Child and Adolescent Mental Health Services (CAMHS) amongst minority ethnic groups compared to their white counterparts [3]. It is important to explore factors and potential barriers faced by young people from ethnic minority backgrounds to address this health inequality. This is especially relevant in the current COVID-19 pandemic, which has prompted the rapid adoption of remote telehealth solutions worldwide, potentially changing the impact of such inequalities. Such services, including virtual clinics have been utilised and studied prior to the pandemic and are overall viewed positively [4].

We hypothesise that telepsychiatry provides the opportunity to revolutionise accessibility to mental health care for young people as it may overcome some of the challenges ethnic minority groups currently face. In this piece, we first discuss the impact of the COVID-19 pandemic on mental health in young people from Afro-Caribbean backgrounds as well as the pre-existing barriers in providing mental health support to these service-users. We also discuss potential opportunities of virtual clinics and health apps compared to face-to-face mental health consultations in engaging with this group of young people.

## IMPACT OF COVID-19 ON MENTAL HEALTH

In England, the differences in prevalence of adult mental health conditions across ethnicities is well-known: the prevalence of psychosis in Afro-Caribbean males at university is 3.1% compared with 0.2% in their white counterparts [5]. These pre-existing ethnic disparities may have been further exacerbated by the COVID-19 pandemic. A survey conducted during July 2020 found that, since 2017, the prevalence of probable mental health disorders has nearly doubled in those from Black, Asian and Minority Ethnic (BAME) backgrounds (5.1% to 10%) and increased by a third in adolescents who identify as white (14.8% to 20.3%), however, the breakdown of BAME was not provided [2]. While the percentage of probable mental health disorders was found to be higher in those who identified as white in both 2017 and 2020, the comparatively larger rate of increase in those from BAME backgrounds is notable, and Clarke et al suggest the latest increases may be linked to the COVID-19 pandemic [2]. Evidence that ethnic minority groups are at increased risk of infection and death may compound racial trauma experienced amongst these communities [6]. Furthermore, BAME communities were greater impacted by issues related to housing, finances, and employment over the pandemic [7]. Therefore, the financial repercussions of the pandemic may have further impacted on the mental well-being of these groups.

## UPTAKE OF MENTAL HEALTH SERVICES

Children from BAME backgrounds are less likely to access CAMHS compared to White British children [3]. Furthermore, the Lambeth Clinical Commissioning Group found that African-Caribbeans account for almost 70% of people detained in secure psychiatric settings despite making up only 26% of the local population [8]. This could be due to failure in accessing support services at a young age as this has been linked to a greater likelihood of involuntary admission to hospital [9]. Studies have found children from ethnic minority groups are more likely to terminate treatment prematurely [3]. Another found that Black children were almost ten times more likely to be referred to Child and Adolescent Mental Health Services (CAMHS) through social services than primary care, compared to White British children [3]. This could be due to these patients being less likely to be registered with a primary care service, or cultural differences in symptom expression leading to misdiagnosis [3]. It is important to understand the factors causing these children to be more likely to access CAMHS via compulsory than voluntary pathways [3], however, among the wider literature, there are limitations in research focussing on barriers faced by Afro-Caribbean adolescents when accessing mental health services. Therefore, when detailing the barriers, this opinion piece refers to a paper by Memon et al, which includes barriers faced by those from Afro-Caribbean backgrounds as some of these may be applicable to adolescents, and reports by the London Assembly Health Committee from 2015 and the Young Hammersmith and Fulham Foundation (YHFF) [1,5,8].

One barrier young people from Afro-Caribbean backgrounds may face in accessing mental health services is the stigma [1,5]. Telepsychiatry has great potential to reduce the impact of stigma as service-users can access services discretely. A survey conducted by the Education Policy Institute found that 17.6% of users of online mental health service, Kooth, reported themselves as BAME [10]. Yet, 10% of the population in the area surveyed are reported as BAME, suggesting that Kooth may have an overrepresentation of young people from ethnic minority backgrounds [10]. This may be due to the anonymity the service provides, thereby reducing service-users’ feelings of shame, related to the stigma of mental illness, in accessing services. However, whilst anonymised mental health services have a high uptake of BAME service-users, this may not be the case in non-anonymised services, such as virtual clinics.

**Figure:**
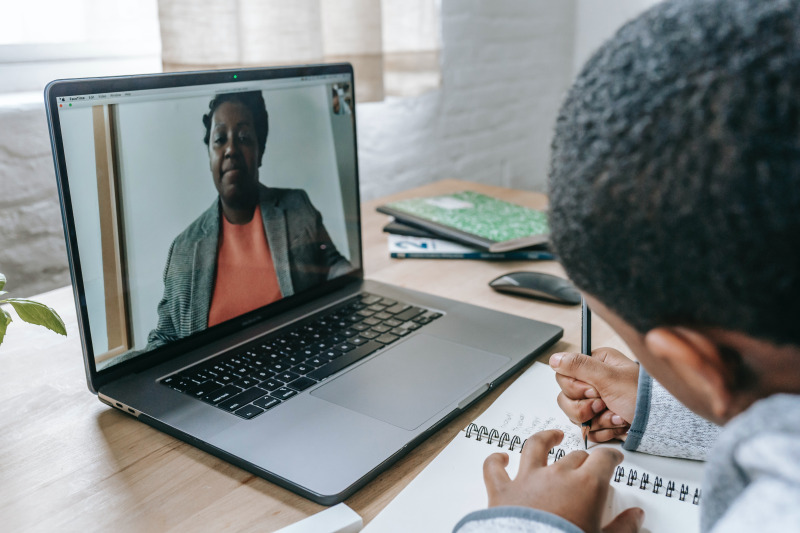
Photo: Videoconferencing as a tool to facilitate discussion remotely in young people from Afro-Caribbean backgrounds (from https://www.pexels.com/, by Katerina Holmes).

The taboo subject of mental health can lead to a lack of awareness of mental health services, as evidenced in the YHFF report which included interviews with adolescents of the African Diaspora [1]. A lack of understanding of mental health disorders was also noted which was associated with limited engagement with services [1]. Telepsychiatry in the form of apps could aid in promoting and raising awareness of mental health disorders as well as awareness of mental health services available within the community to better inform young people wishing to access them.

Another identified barrier young people from Afro-Caribbean backgrounds may face is not being understood by service providers [5], instead sometimes preferring to seek support from systems such as religious or family networks [8]. Telepsychiatry could provide an opportunity to connect health services with these established functioning support networks [8], with potential to strengthen the doctor-patient relationship. Telepsychiatry can also help provide an integrated model for mental health, incorporating diverse multi-disciplinary teams in the assessment and diagnosis, pharmacological and psychosocial services, and follow-up or home care via one, holistic medium [11]. Furthermore, there is improved quality of care in children and adolescents and increasing usage among ethnic minority populations [11].

With regards to patients not feeling understood by service providers, there is research exploring the use of ethnic matching between patient and clinician. According to a literature review of studies with adults, culturally adapted psychotherapies (which can be utilised in the management of emotional disorders) and telepsychiatry that included ethnic matching showed benefit [2]. Telepsychiatry would enable psychiatrists to connect from geographically different locations to their patients, and could be a solution to the lack of diversity among mental health professionals that has been cited [1]. Conversely, this would be difficult to implement in reality. Furthermore, young males of the African Diaspora reportedly felt that differing ethnicity, gender or socioeconomic position between patient and health care professional did not create an instant barrier [1]. A more sustainable approach may involve increasing communication skill training to better equip health care professionals to understand their patients even if they may not share the same cultural values. This is particularly important as cultural constructs of mental health can influence a patient’s perception of their illness and recovery process [12]. Therefore, a lack of cultural competency may adversely impact on the support provided from clinicians to patients of different ethnic backgrounds.

An imbalance of power between provider and service users from Afro-Caribbean backgrounds has also been identified [5], with users feeling as though they were being “talked down to” by providers [5]. A scoping review identified that adolescents find videoconferencing promotes transfer of power and allows them to feel more comfortable about terminating the consultation or walking out [13]. Therefore, telepsychiatry help strengthen the doctor-patient relationship and make care more focussed towards the patients wants and needs. However, a shortcoming of telepsychiatry may be inequitable access and the creation of a “digital divide” [13]. This is something which should be explored further as may result in the creation of additional barriers vulnerable groups may face.

## CONCLUSION

COVID-19 has highlighted inequalities in health amongst ethnicities. It is important that the impact on mental health across different ethnicities is monitored over time to better understand the impact COVID-19 has had on mental health. Overall, the rapid, widespread adoption of telehealth could be beneficial in reducing barriers between mental health service providers and Afro-Caribbean adolescent users. We found limited published research into barriers faced by specific ethnic groups, with many grouped together using the term ‘BAME’. Further investigation exploring this and stratifying by ethnicity should be welcomed to generate evidence to ensure targeted recommendations for improving mental health service provision. COVID-19 has left widespread impact on physical health and mental well-being and it is vital that services are equipped to deliver support best suited to the service-user needs.
